# Supplementary material for: Bacteriological analysis and antibiotic resistance in patients with diabetic foot ulcers in Dhaka
Source: PLoS One. 2024 May 17;19(5):e0301767. doi: 10.1371/journal.pone.0301767 (PMC11101115; doi:10.1371/journal.pone.0301767)
Supplement: S1 Table — (DOCX) [file pone.0301767.s001.docx]

| **Wagner's Classification** | **Description** |
| --- | --- |
| 0 | Skin intact, but bony deformities lead to "foot at risk" |
| 1 | Superficial ulcer |
| 2 | Deeper, full-thickness extension |
| 3 | Deep abscess formation or osteomyelitis |
| 4 | Partial Gangrene of forefoot |
| 5 | Extensive Gangrene |
