# Supplementary material for: Bacteriological analysis and antibiotic resistance in patients with diabetic foot ulcers in Dhaka
Source: PLoS One. 2024 May 17;19(5):e0301767. doi: 10.1371/journal.pone.0301767 (PMC11101115; doi:10.1371/journal.pone.0301767)
Supplement: S2 Table — (DOCX) [file pone.0301767.s002.docx]

| **Organism** | **Gram Positive/Negative** | **Media** | **Expected Colony Morphology** |
| --- | --- | --- | --- |
| *Staphylococcus aureus* | Gram-positive | MSA agar | Yellow/white colonies surrounded by the yellow zone |
| *Pseudomonas aeruginosa* | Gram-negative | Cetrimide Agar | Yellow-green glow under UV ray |
| *Klebsiella pneumoniae* | Gram-negative | HiCrome KPC Agar (without added antibiotic) | Bluish-green |
| *Escherichia coli* | Gram-negative | HiCrome UTI Agar (without added antibiotic) | Blue-Purple colonies |
| *Escherichia coli* | Gram-negative | EMB Agar | Purple with a black center and green metallic sheen |
| *Proteus spp.* | Gram-negative | MacConkey Agar | Colorless colonies surrounded by the orange zone |
