# Supplementary material for: Bacteriological analysis and antibiotic resistance in patients with diabetic foot ulcers in Dhaka
Source: PLoS One. 2024 May 17;19(5):e0301767. doi: 10.1371/journal.pone.0301767 (PMC11101115; doi:10.1371/journal.pone.0301767)
Supplement: S3 Table — * Hemolysis and coagulase test was performed on Staphylococcus aureus isolates for further confirmation of the isolates. & To confirm the identity of Escherichia coli isolates, they were subjected to additional subculturing on EMB Agar, and their colony morphology was observed. # To confirm the identity of Proteus spp. isolates, they were subjected to additional subculturing on Blood Agar, allowing for the observation of swarming colonies. (DOCX) [file pone.0301767.s003.docx]

| **Organism** | **Catalase** | **Oxidase** | **Glucose**  **Ferm.** | **Sucrose**  **Ferm.** | **Lactose**  **Ferm.** | **Gas Prod.** | **H_2_S**  **Prod.** | **Motility** | **Indole** | **Urease** | **MR** | **VP** | **Citrate utilization** |
| --- | --- | --- | --- | --- | --- | --- | --- | --- | --- | --- | --- | --- | --- |
| *Staphylococcus aureus** | +ve | -ve | +ve | +ve | +ve | -ve | -ve | -ve | -ve | +ve | +ve | +ve | +ve |
| *Escherichia coli^&^* | +ve | -ve | +ve | variable | +ve | +ve | +ve | +ve | +ve | -ve | +ve | -ve | -ve |
| *Pseudomonas aeruginosa* | +ve | +ve | -ve | -ve | -ve | +ve | -ve | +ve | -ve | -ve | -ve | -ve | +ve |
| *Klebsiella pneumoniae* | +ve | -ve | +ve | +ve | +ve | +ve | -ve | -ve | -ve | +ve | -ve | +ve | +ve |
| *Proteus spp.#* | -ve | +ve | +ve | -ve | -ve | +ve | +ve | +ve | +ve | +ve | +ve | -ve | -ve |
