# Supplementary material for: Bacteriological analysis and antibiotic resistance in patients with diabetic foot ulcers in Dhaka
Source: PLoS One. 2024 May 17;19(5):e0301767. doi: 10.1371/journal.pone.0301767 (PMC11101115; doi:10.1371/journal.pone.0301767)
Supplement: S4 Table — (DOCX) [file pone.0301767.s004.docx]

| **Name of bacteria** | **Primer Designation** | **Primer Sequence (5′to 3′)** | **Product size** | **PCR conditions** |
| --- | --- | --- | --- | --- |
| *Klebsiella pneumoniae* | KP Pf-F: | ATTTGAAGAGGTTGCAAACGAT | 130 bp | The cycling conditions were 10 min at 94°C followed by 35 cycles of 30 s at 94°C, 20 s at 57°C and 20 s at 72°C, followed by a 10 min hold at 72°C. |
|  | KP Pr1-R: | TTCACTCTGAAGTTTTCTTGTGTTC |  |  |
| *Escherichia coli* | ECO- F | GACCTCGGTTTAGTTCACAGA | 585 bp | Initial denaturation at 95°C for 5 min; 35 cycles of denaturation at 94°C for 45s, annealing at 45°C for 45s, and extension for 1 min followed by a final extension at 72°C for 5 min. |
|  | ECO-R | CACACGCTGACGCTGACCA |  |  |
| *Pseudomonas*  *aeruginosa* | PA-SS-F | GGGGGATCTTCGGACCTCA | 956 bp | Initial denaturation at 95°C for 2 min; 25 cycles of denaturation at 94°C for the 20s, annealing at 58°C for 20s, elongation at 72°C for 40s, final extension at 72°C for 1min. |
|  | PA-SS-R | TCCTTAGAGTGCCCACCCG |  |  |
| *Staphylococcus aureus* | Sa442-F | AATCTTTGTCGGTACACGATATTCTTCACG | 108 bp | 3 min at 96°C and then 30 cycles of 1 s at 95°C for the denaturation step and 30 s at 55°C for the annealing-extension step. |
|  | Sa442-R | CGTAATGAGATTTCAGTAGATAATACAACA |  |  |
