# Supplementary material for: Bacteriological analysis and antibiotic resistance in patients with diabetic foot ulcers in Dhaka
Source: PLoS One. 2024 May 17;19(5):e0301767. doi: 10.1371/journal.pone.0301767 (PMC11101115; doi:10.1371/journal.pone.0301767)
Supplement: S5 Table — (DOCX) [file pone.0301767.s005.docx]

| **Antibiotic** | **Group** | **Effective against** | **Disc code** | **Disc potency (µg)** | **Interpretative Criteria** | | |
| --- | --- | --- | --- | --- | --- | --- | --- |
|  |  |  |  |  | **Sensitive mm or more** | **Intermediate mm** | **Resistant mm or less** |
| Azithromycin | Macrolide | Gram-positive and Gram-negative | AZM | 15 | 18 | 14–17 | 13 |
| Amikacin | Aminoglycoside | Gram-positive and Gram-negative | AK | 30 | 17 | 15–16 | 14 |
| Colistin | Polymyxin E | Gram-negative | CT | 10 | - | 11-17 | - |
| Cefepime | Cephalosporin | Gram-positive and Gram-negative | CPM | 30 | 25 | 19–24 | 18 |
| Meropenem | Carbapenem | Gram-positive and Gram-negative | MEM | 10 | 23 | 20–22 | 19 |
| Imipenem | Carbapenem | Gram-positive and Gram-negative | IMI | 10 | 23 | 20–22 | 19 |
| Piperacillin/ Tazobactam | Penicillin and beta-lactamase inhibitor | Gram-positive and Gram-negative | PIT | 100/10 | 21 | 18–20 | 17 |
| Streptomycin | Aminoglycoside | Gram-positive and Gram-negative | S | 10 | 15 | 12-14 | 11 |
| Ampicillin | Beta-lactamase | Gram-positive and Gram-negative | AMP | 10 | 17 | 14–16 | 13 |
| Norfloxacin | Fluoroquinolone | Gram-positive and Gram-negative | NX | 10 | 17 | 13-16 | 12 |
| Vancomycin | Glycopeptide | Gram-positive | VA | 30 | 17 | 15–16 | 14 |
| Linezolid | Oxazolidinones | Gram-positive | LZ | 30 | 23 | 21–22 | 20 |
| Tigecycline | Glycylcyline | Gram-positive and Gram-negative | TGC | 15 | 18 | 15-17 | 15 |
