# Supplementary figures and images for: Bacteriological analysis and antibiotic resistance in patients with diabetic foot ulcers in Dhaka
Source: PLoS One. 2024 May 17;19(5):e0301767. doi: 10.1371/journal.pone.0301767 (PMC11101115; doi:10.1371/journal.pone.0301767)

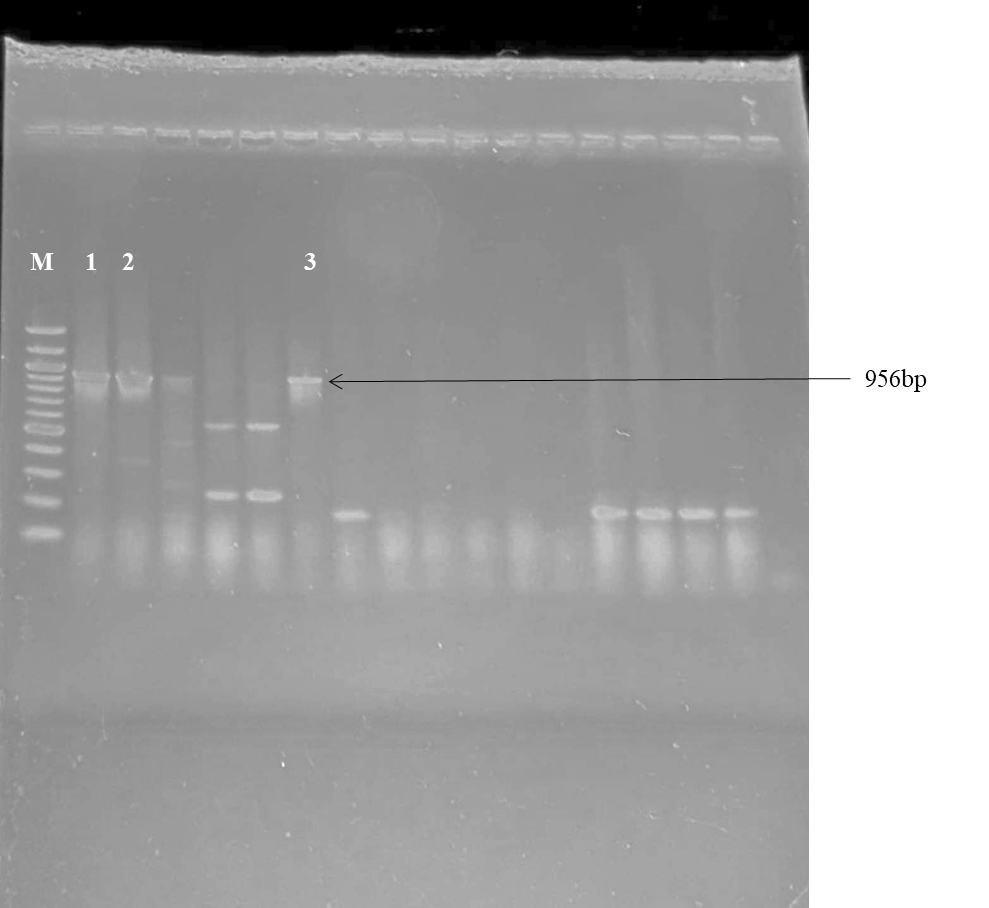

Supplement: S1 Fig — Here, Lane M is 100 bp DNA marker, and Lane 1–3 are some positive samples at 956 bp. The other bands did not show the correct band size and therefore not included in our positive result. (TIF) [file pone.0301767.s006.tif]

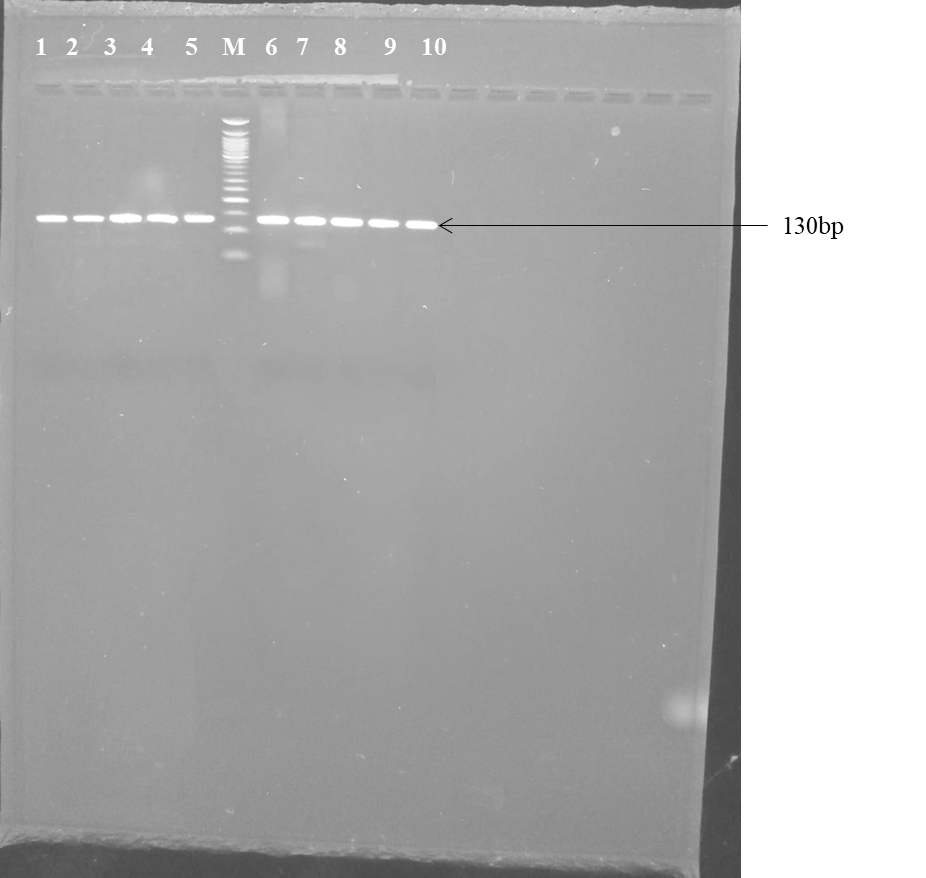

Supplement: S2 Fig — Here, Lane M is 50 bp DNA marker, and Lane1-10 are some positive samples at 130 bp. (TIF) [file pone.0301767.s007.tif]

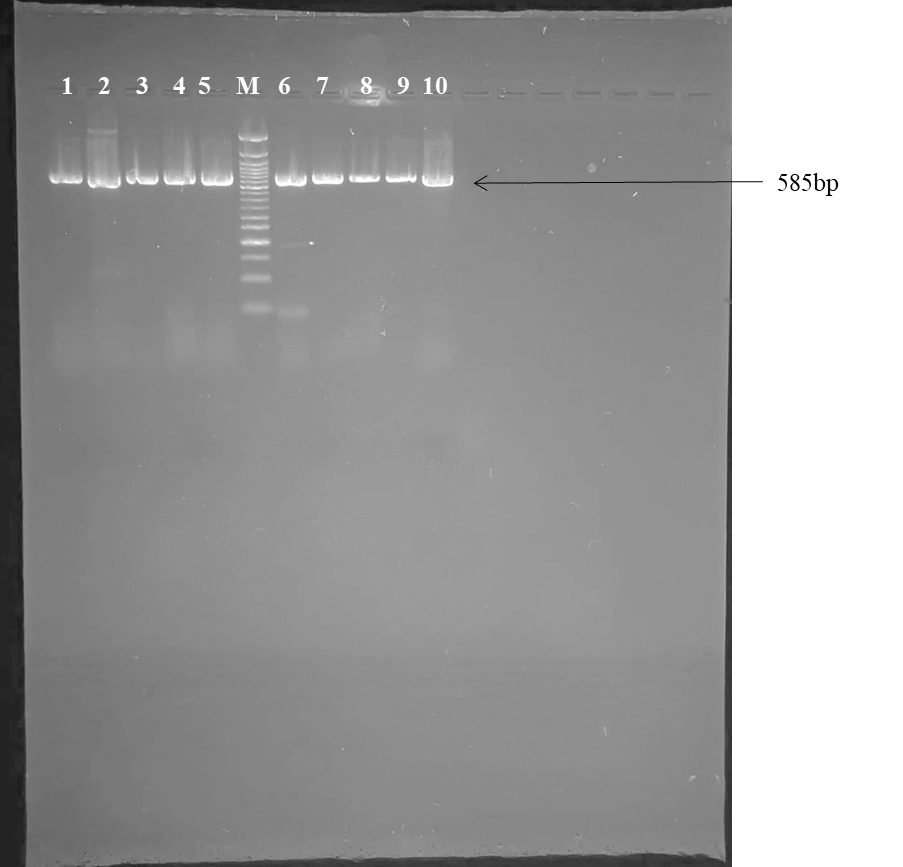

Supplement: S3 Fig — Here, Lane M is 50 bp DNA marker, and Lane 1–10 are some positive samples at 585bp. (TIF) [file pone.0301767.s008.tif]

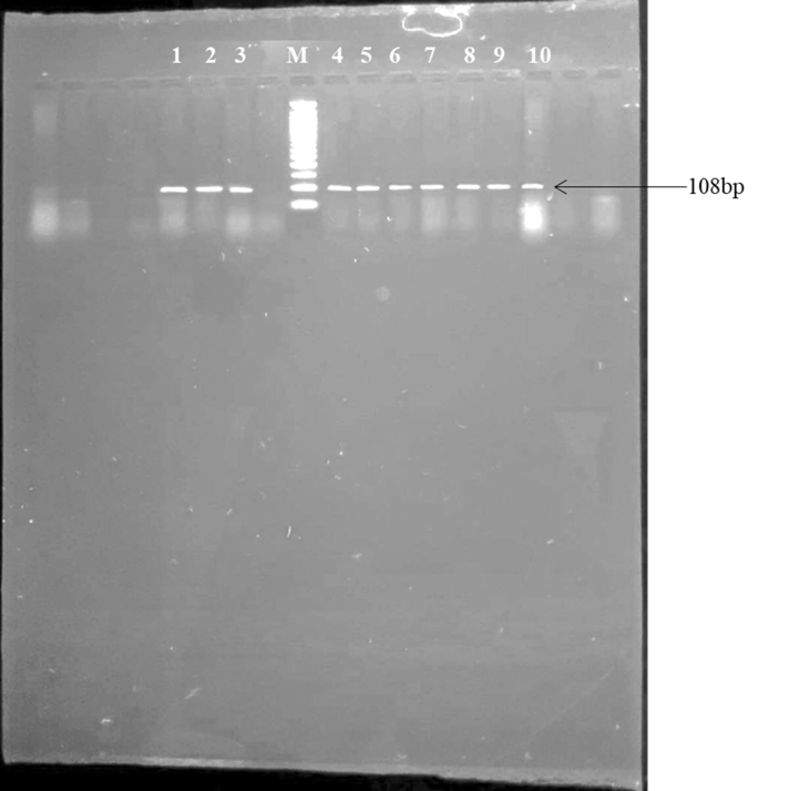

Supplement: S4 Fig — Here Lane (M) DNA is 50 bp marker, and Lane (1–10) are some positive samples at 108bp. (TIF) [file pone.0301767.s009.tif]
