# Supplementary material for: Bacteriological analysis and antibiotic resistance in patients with diabetic foot ulcers in Dhaka
Source: PLoS One. 2024 May 17;19(5):e0301767. doi: 10.1371/journal.pone.0301767 (PMC11101115; doi:10.1371/journal.pone.0301767)
Supplement: S1 Raw images — (PDF) [file pone.0301767.s010.pdf]

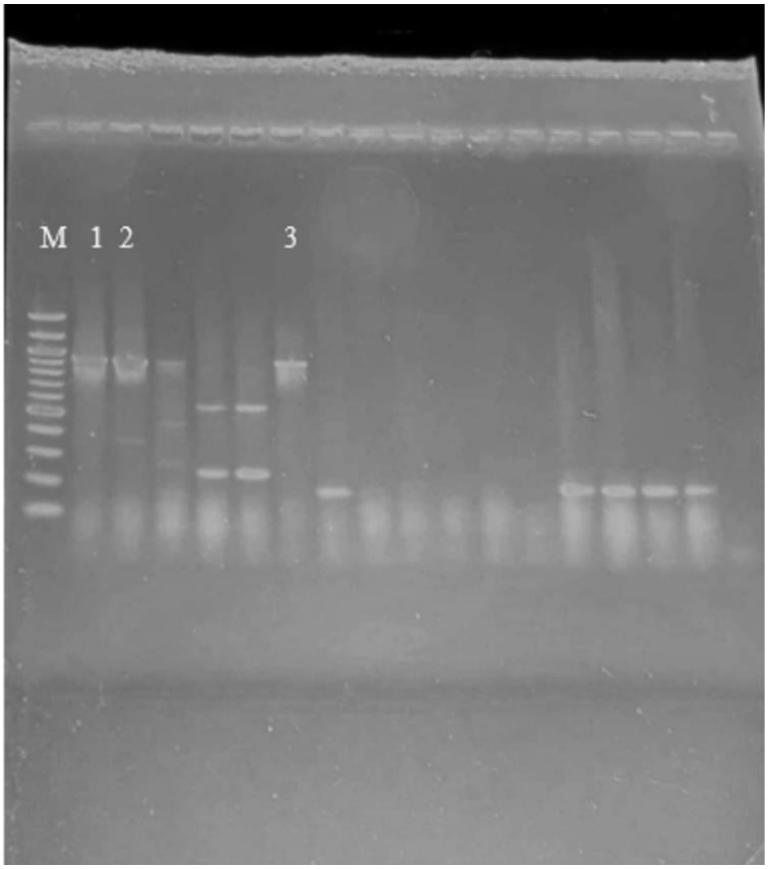

S1 Fig. Agarose gel electrophoresis of PCR assay of *Pseudomonas aeruginosa* isolates. Here, Lane M is 100 bp DNA marker, and Lane 1-3 are some positive samples at 956 bp. The other bands did not show the correct band size and therefore not included in our positive result. The image was captured using a camera while the agarose gel was positioned under a UV transilluminator. The complete original image was utilized to generate the figure panel.

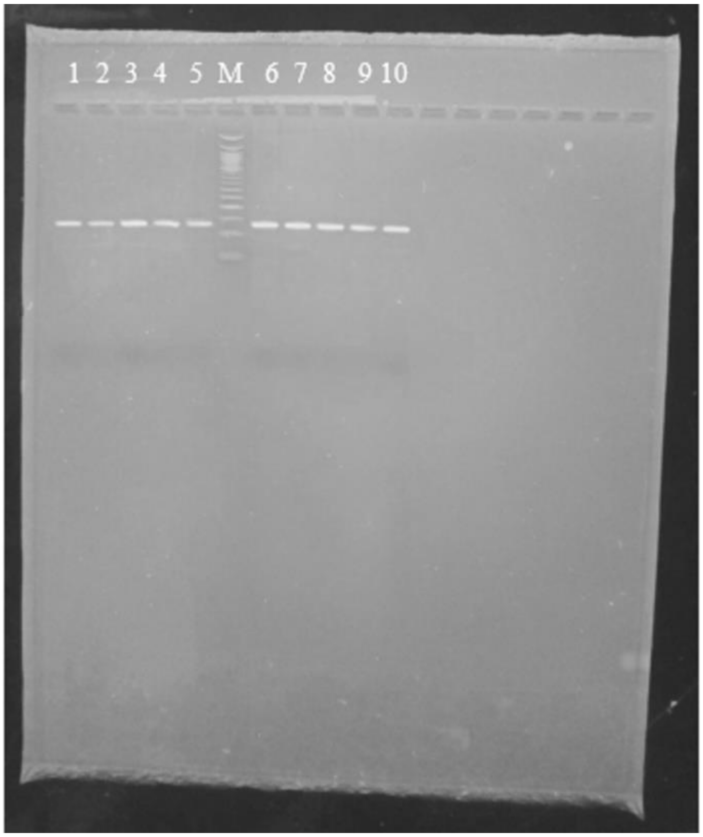

S2 Fig. Agarose gel electrophoresis of PCR assay of *Klebsiella pneumoniae* isolates. Here, Lane M is 50 bp DNA marker, and Lane1-10 are some positive samples at 130 bp. The image was captured using a camera while the agarose gel was positioned under a UV transilluminator. The complete original image was utilized to generate the figure panel.

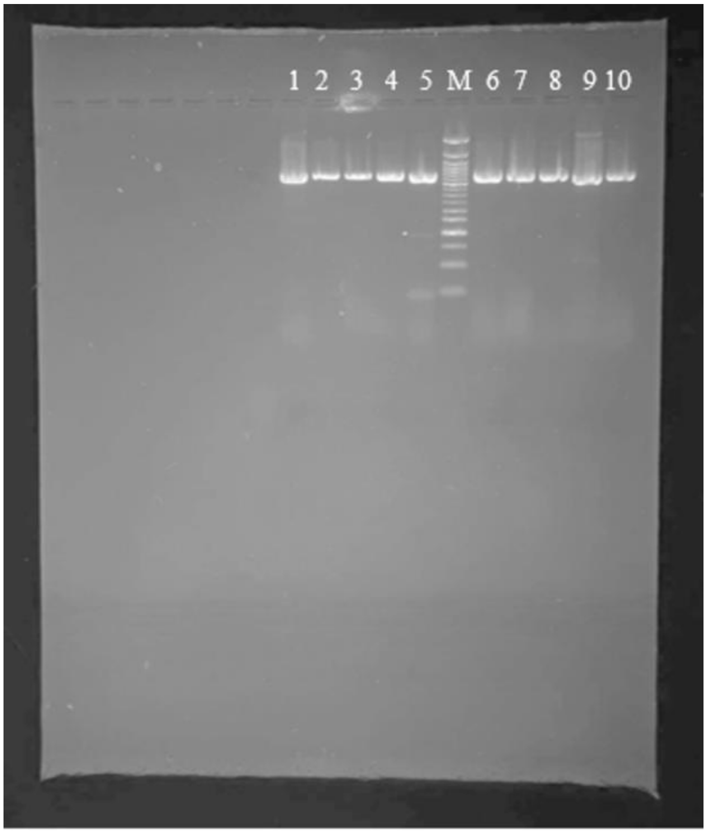

S3 Fig. Agarose gel electrophoresis of PCR assay of *Escherichia coli* isolates. Here, Lane M is 50 bp DNA marker, and Lane 1-10 are some positive samples at 585bp. The image was captured using a camera while the agarose gel was positioned under a UV transilluminator. The complete original image was utilized to generate the figure panel.

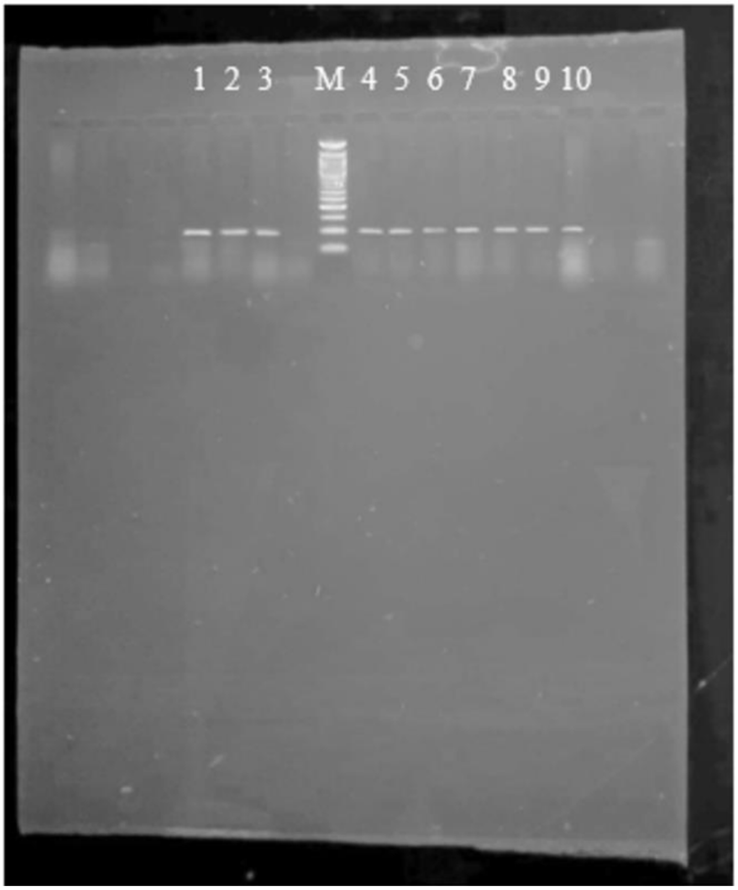

S4 Fig. Agarose gel electrophoresis of PCR assay of *Staphylococcus aureus* isolates. Here Lane (M) DNA is 50 bp marker, and Lane (1-10) are some positive samples at 108bp. The image was captured using a camera while the agarose gel was positioned under a UV transilluminator. The complete original image was utilized to generate the figure panel.
