# Supplementary material for: Bacteriological analysis and antibiotic resistance in patients with diabetic foot ulcers in Dhaka
Source: PLoS One. 2024 May 17;19(5):e0301767. doi: 10.1371/journal.pone.0301767 (PMC11101115; doi:10.1371/journal.pone.0301767)
Supplement: Attachment — Submitted filename: Reviewer Responses_PLOS ONE.docx [file pone.0301767.s011.docx]

Reviewer responses:

The authors carried out this study on an important and most studied disease like diabetes. They have conducted this research on people who have backgrounds in Bangladesh. The author's main study focus was on the diabetic ulcers and the bacterial isolates that are colonized, and the study was conducted more than the required relevance to socioeconomic and educational backgrounds. The authors give a lot of examples and more over frequently they are trying to justify their findings with the previous data and depend on survey studies conducted by the national and international diabetic organizations/ foundations. After reading, understanding and looking at their research results and discussions, I have a few major and minor comments on this manuscript.

Major concerns:

1. The authors could have taken up a larger demographics rather than a small number of cohorts. That could help to generate a wider bacterial colony to identify and test their antibiotic resistance in vitro conditions.
2. The authors could have focused on the host and the identified bacterial species and their specific relation in the background of diabetes, as the authors claimed they recruited people from the urban and rural areas which might include hygienic and unhygienic lifestyles might become non-specific hosts for several bacteria for a short period. Precisely identifying the diabetic ulcerative bacterial populations will provide a valuable clue for the treatment.
3. The authors seem to have the same kind of growth media to culture different species of bacteria such as gram-positive and gram-negative and also we cannot rule out the bacterial adaptations to the available nutrients that potentially cause response or resistance to different antibiotics.
4. I would suggest the authors conduct more deliberate experiments to see the bacterial responses to an antibiotic, the current study involved basic experimental settings instead of deep investigations.

Minor concerns:

1. The authors have missed a great option to show their all data and importantly they have limitedly presented their results and focused to discuss on the already available data from various sources to justify their study.
2. The figures have a very poor resolution and are not able to read the Figure 1 contents at all.

Impression:

This study will help as a little reference for further studies whoever wants to investigate deep into the diabetic ulcerative bacteria in connection with antibiotic resistance.
